# The impact of incentive scheme on rural healthcare workforce availability: a case study of Kazakhstan

**DOI:** 10.1186/s12960-024-00905-0

**Published:** 2024-04-11

**Authors:** Bagym Jobalayeva, Zaituna Khismetova, Natalya Glushkova, Zhanat Kozhekenova, Akerke Abzaliyeva, Duman Berikuly, Yuliya Semenova

**Affiliations:** 1 Department of Public Health, JSC “Semey Medical University”, Semey, Republic of Kazakhstan; 2 Department of Epidemiology, Biostatistics & Evidence Based Medicine, Al-Farabi Kazakh National University, Almaty, Republic of Kazakhstan; 3 Department of Public Health, JSC “Asfendiyarov Kazakh National Medical University”, Almaty, Republic of Kazakhstan; 4 Department of Public Health and Social Sciences, “Kazakhstan School of Public Health”, Kazakhstan’s Medical University, Almaty, Republic of Kazakhstan; 5 Deputy Chairperson of the Board for Clinic and Postgraduate Education, JSC “Semey Medical University”, Semey, Republic of Kazakhstan; 6 Nazarbayev University School of Medicine, Astana, Republic of Kazakhstan

**Keywords:** Rural health, Nurses, Physicians, Incentive scheme, Interrupted time series analysis, Kazakhstan

## Abstract

**Background:**

During the 1990–2000, Kazakhstan experienced a decline in the number of healthcare professionals working in rural areas. Since 2009, the national government has been implementing financial incentives to encourage healthcare professionals to relocate to rural areas. This study aims to investigate the temporal and spatial patterns in the distribution of the rural healthcare workforce and evaluate the impact of this incentive scheme.

**Methods:**

Interrupted Time Series Analysis using ARIMA models and Difference in Differences analyzes were conducted to examine the impact of the incentive scheme on the density of different categories of the healthcare workforce in rural Kazakhstan in the period from 2009 to 2020.

**Results:**

There was a significant increase in the number of rural healthcare professionals from 2009 to 2020 in comparison to the period from 1998 to 2008. However, this increase was less pronounced in per capita terms. Moreover, a decline in the density of internists and pediatricians was observed. There is substantial variation in the density of rural nurses and physicians across different regions of Kazakhstan. The incentive scheme introduced in 2009 by the government of Kazakhstan included a one-time allowance and housing incentive. This scheme was found to have contributed insignificantly to the observed increase in the number of rural healthcare professionals.

**Conclusion:**

Future research should be undertaken to examine the impact made by the incentive scheme on other medical subspecialties, particularly primary practitioners. Addressing the shortage of healthcare workers in rural areas is a complex issue that requires a multifaceted approach. Aside from financial incentives, other policies could be considered to increase relocation and improve the retention of healthcare professionals in rural areas.

## Introduction

The provision of healthcare services is a fundamental human right that should be accessible to all individuals, irrespective of their geographic location or socioeconomic status [1]. However, rural areas frequently encounter insufficient resources and staffing shortages, posing challenges in meeting the healthcare needs of their populations. The scarcity of healthcare professionals presents a significant obstacle for rural communities, impeding their right to health.

Strategies such as recruiting students from rural backgrounds and providing comprehensive rural training during their education can significantly contribute to retaining healthcare professionals [2]. Similarly, supporting rural healthcare professionals by financing an advancement of their skills and qualifications has proven effective [2]. However, interventions reliant on regulatory measures, such as mandatory rural service in exchange for licensure or visa waivers, tend to be less effective in retaining professionals beyond their mandatory service period [2]. In contrast to the above-listed interventions, interventions requiring professionals to repay loans upon completion of their rural service demonstrate greater efficacy in terms of retention in rural areas [2]. Overall, prioritizing rural students and offering rural training programs yield better results compared to coercive measures, such as requiring return-of-service for access to professional licenses or provider numbers [2].

Other potential interventions focusing on improved retention in rural areas, such as increasing pay rates and offering financial incentives, can enhance the appeal of medical professionals for working in rural healthcare facilities [3]. Investing in infrastructure and technology can also contribute to making rural primary healthcare practice more attractive [4]. Telemedicine, for instance, provides a viable solution for delivering healthcare services remotely, facilitating easier access to primary healthcare for patients in distant areas [5]. Furthermore, investment in the construction of new medical facilities and the upgrading of existing ones can play a crucial role in attracting and retaining healthcare professionals in rural areas [6, 7].

### Health workforce in rural Kazakhstan and the incentive scheme

Kazakhstan, a former member of the Union Soviet Socialist Republics (USSR), is a Central Asian country that gained independence in 1991. During the Soviet period, the Semashko model of healthcare was in place, the legacy of which continues to influence the organization and governance of the healthcare system to this day. The Ministry of Health (MoH) plays a major role in managing the healthcare system, shaping policies and strategies, which largely target the primary healthcare sector. Under the Semashko model, primary care for urban residents was provided via a network of polyclinics, staffed by internists, pediatricians, and other specialists. In rural areas, healthcare services were mostly provided by Feldsher/Midwife Health Posts (FHPs) in small-scale villages and by polyclinics in larger rural settings, with similar medical personnel to those in urban areas. They were responsible for providing immediate, primary care and emergency ambulance services to the population in need, as well as health promotion. After gaining independence, Kazakhstan initiated healthcare system reforms, transforming polyclinics into Family Medical Centers (FMCs), thus expanding the primary healthcare network in urban areas. However, this led to a reduction in the density of healthcare networks in rural areas and the migration of healthcare professionals to urban areas [8].

During the early years of transition, many government healthcare facilities were closed, and private facilities were opened, affecting both urban and rural healthcare facilities. Approximately 12% of FMCs are now private, providing services funded by public funds. Reforms also included a shift to capitation for primary healthcare services, but rural healthcare remained underfinanced, with about 40% of the Kazakhstani population residing in rural areas [9]. In response to the observed deficiency in public health funds, an obligatory health insurance system was introduced, although out-of-pocket payments for primary healthcare services continue to play a role both in urban and rural areas [9].

Due to the ongoing emigration of medical professionals to urban centers and the consequent decline in the rural healthcare network, Kazakhstan recognized the pressing need to address this emerging public health issue [10]. Two national projects, namely the “Construction of 350 Family Medical Centers, Feldsher-Midwife Health Posts, and Polyclinics,” and the “Construction of 100 Schools, 100 Hospitals,” were implemented between 2008 and 2016 to bolster the network of healthcare facilities in rural areas by constructing modern FMCs, FHPs, polyclinics, and hospitals, resulting in an increase in the density of rural healthcare facilities [10]. However, despite the availability of nine medical schools, addressing the shortage of rural medical personnel has remained a persistent challenge due to the reluctance of medical school graduates to pursue rural careers [10].

The Observatory of Human Resources for Health (OHRH) was established by the MoH order in 2012, with the mandate to collect data, monitor, plan, and forecast the MoH’s needs for the health workforce in both urban and rural areas. Since the majority of medical students in Kazakhstan are trained at the expense of public funds, the data collected by the OHRH are utilized to determine the number of students requiring training at public expense [11]. Beginning in 2009, the national government instituted an incentive scheme for all types of professionals willing to relocate to rural areas, which remains operational. No caps were set for the number of professionals eligible for the scheme, and it is open to professionals with both university and college degrees. This scheme involves financial remuneration in the form of a one-time allowance, approximately equivalent to one monthly salary. Additionally, the provision of a low-rate loan for the purchase of housing is envisaged. Despite a thorough search for information available in open access, evidence indicating an evaluation of the impact of this incentive scheme by the OHRH was not found. Therefore, while a policy aimed at augmenting the availability of healthcare personnel in rural areas has been enacted, its efficacy remains incompletely assessed. Given that public funds were employed to subsidize this financial scheme and the country budget experiences shortages, reconsideration of public expenses is warranted [9]. Currently, there is a lack of estimates on rural population health needs, including premature mortality, prevalence of emergencies, risk behaviors, and other unmet health needs, but according to official statistics, the health indicators of the rural population in Kazakhstan are non-inferior to those of urban areas [9]. Similarly, there is a lack of data on the amount of public money that has been spent on the incentive scheme, how many people have applied and used the scheme, and the number of people and reasons for not receiving the scheme. As of 2024, the scheme has been in existence for 15 years.

### Study aim

The aim of this study is to utilize official statistical data to assess the impact of this incentive scheme on the numbers and density of healthcare personnel in rural areas in Kazakhstan. Additionally, this study reports on temporal and spatial trends in the distribution of various types of healthcare professionals across the country.

## Methods

### Study design

The present study utilized a retrospective design and relied on official statistical data released by the MoH. Additionally, population data for Kazakhstan were obtained from the Bureau of National Statistics (BNS). To collect information on the incentives offered to healthcare professionals practicing in rural areas, we utilized the Adilet database, which is the official database of the Ministry of Justice Kazakhstan that provides unrestricted access to all legislative acts enacted in the country since gaining independence in 1991. We conducted searches in both Russian and Kazakh languages, utilizing the search terms “льгoты” (incentives) OR “пoддepжкa” (support) OR “выплaты” (payments) AND “cпeциaлиcты здpaвooxpaнeния” (healthcare professionals) AND “ceльcкиe нaceлeнныe пyнкты” (rural settlements) for Russian, and “apтықшылықтap” (incentives) OR “қoлдay” (support), OR “төлeмдep” (payments) AND “дeнcayлық caқтay мaмaндapы” (healthcare professionals), AND “ ayылдық eлдi мeкeндep” (rural settlements) for Kazakh.

We analyzed all legislative acts related to the provision of incentives to healthcare professionals practicing in rural areas and compiled a table displaying the range and size of benefits included in the scheme. To enhance clarity for the international scientific community, we converted the amounts expressed in Kazakhstani Tenge into US dollars utilizing the corresponding year's exchange rates [12].

### Study units

The study examined the number of medical doctors (physicians) and nurses working in rural areas of the country during the period from 1998 to 2020. It is common practice for the MoH in Kazakhstan to report on the number of nurses along with the number of dentists, as many of them do not possess higher medical education. Additionally, aside from the overall number of physicians, the MoH presents data on various medical subspecialties. We extracted data on those subspecialties for which information was available for the entire study period, including neurosurgeons, cardiologists, trauma specialists, endocrinologists, radiologists, neurologists, urologists, anesthesiologists, general surgeons, otorhinolaryngologists, ophthalmologists, obstetrician–gynecologists, dentists, internists, and pediatricians. The number of rural primary practitioners is reported only from 2009 onward; therefore, data for this group were extracted for the period from 2009 to 2020, while data for other subspecialties were extracted for the period from 2001 to 2020.

To calculate the density of the healthcare workforce, we referred to the website of the Global Health Observatory, World Health Organization (WHO) [13]. The density of physicians working in rural areas was computed per 10,000 population using the following formula:

$$\text{Density of rural physicians}\hspace{0.17em}=\hspace{0.17em}\text{number of rural physicians}/\text{midyear number of rural population }*\mathrm{ 10,000}.$$  

Similarly, the density of urban physicians was calculated per 10,000 urban population. Additionally, the density of rural nursing personnel was computed per 10,000 population using the following formula:

$$\text{Density of rural nurses}\hspace{0.17em}=\hspace{0.17em}\text{number of rural nurses}/\text{midyear number of rural population }*\mathrm{ 10,000}.$$  

The MoH typically issues a national statistical compilation on key healthcare indicators, which is made public on the website of the Republican Center for Health Development [14]. The published data comprise information on the healthcare network and personnel, which are disaggregated by the region (oblast) and location (urban versus rural) of the country. The earliest compilation was issued in 1998, and the latest available compilation is dated 2020. The BNS provided data on the midyear population obtained from their annual statistical compilations [9], which are presented in disaggregation by place of residence (urban vs. rural) and the number of rural populations. We relied solely on the number of rural populations, and the data disaggregated by region were also obtained by us to calculate density indicators.

### Statistical analysis

We utilized the Statistical Package for Social Sciences (IBM SPSS Statistics) version 24.0 to perform all statistical tests. The interrupted time series analysis was carried out using an autoregressive integrated moving average (ARIMA) modeling approach. We first set the annual frequency for all analyses and plotted graphs of the data on the number of healthcare personnel against time. The resulting sequence charts were examined for stationarity by computing the autocorrelation (ACF) and partial autocorrelation (PACF) charts. Since the data were nonstationary, we applied first differencing and plotted the ACF and PACF charts again to observe the remaining autocorrelations. We counted the number of significant lags to determine the value of autocorrelation (p) and moving average (q) needed to make all lags insignificant [15]. The Difference in Differences (DID) analysis was performed by means of running a linear regression with urban healthcare personnel serving as a control group.

All physicians’ and nurses' total numbers were included in the models, and we also included disaggregation by the type of major specialty (all types of internal medicine subspecialties and all types of surgery specialties), as provided by the MoH. We selected 2009, the year the benefits package was introduced, as the year of intervention and the percentage point change (PPC) was calculated for interrupted time series analysis, while unstandardized beta coefficient (β) was calculated for the DID analysis. All tests were considered significant at *p* = 0.05.

To visualize the 2008 and 2020 regional density rates for all physicians and nurses per 10,000 population, we constructed maps of Kazakhstan with the help of QGIS 3.26 “Buenos Aires”.

## Results

In 2009, the Kazakhstani Government enacted a decree offering an incentive scheme for professionals relocating to rural areas. This package is expressed in the Monthly Calculation Index (MCI), a unit used in Kazakhstan to calculate taxes, penalties, and benefits [16]. According to the decree, eligible professionals received a one-time allowance of 70 MCIs, which was roughly equivalent to 750 USD, and a housing incentive of 600 MCIs, which approximated 6,425 USD [17]. Over time, this decree underwent several amendments, including an increase in the size of both the one-time allowance and housing incentive [18, 19]. However, the value of the MCI is subject to annual revision by the government, resulting in an increase in the size of allowances paid in Tenge [16]. Unfortunately, the Kazakhstani Tenge experienced several devaluations during the same time period [20], resulting in a reduction in the dollar equivalents of the incentive scheme.

The housing incentive is a bank loan offered at an incredibly favorable rate of 0.01% per annum, which can be repaid over a period of up to 15 years. However, if the employment relationship is terminated before 3 years, the budget funds are returned to the government in full. Table 1 provides insight into the incentive scheme offered to Kazakhstani professionals moving to rural areas from 2009 to the present day.

**Table 1:** An overview of the incentives provided to specialists practicing in rural areas, as outlined in government decrees

Government Decree, reference	One-time allowance, MCI (Tenge/US dollars)	Housing incentive, MCI* (Tenge/US dollars)
Government Decree dated 2009 [17]	70 (90,720/749.6)	600 (777,600/6,425.4)
Amendments to the Decree implemented in 2011 [18]	70 (105,840/729.0)	1500 (2,268,000/15,620.9)
Amendments to the Decree implemented in 2019 [19]	100 (252,500/674.8)	1500 (3,787,500/10,122.1)

*MCI, Monthly calculation index

Despite conducting a careful and comprehensive search, we were unable to identify incentives specifically designed to retain health professionals in rural areas, apart from the housing incentive. Therefore, it can be concluded that the emphasis is primarily placed on the relocation of healthcare professionals rather than on their retention in rural areas.

To assess the impact of the incentive scheme implemented in 2009 on the healthcare workforce numbers in rural areas of Kazakhstan, we conducted a series of interrupted time series analyses. Table 2 presents the most appropriate ARIMA models, along with the computation of stationary R^2^ and estimates of PPC resulting from the intervention and the observed p values. Our findings indicate a significant increase in the number of nurses, followed by all physicians, whereas the number of subspecialties in internal medicine and surgery saw a minor increase. However, none of the changes observed were statistically significant, and the observed stationary R^2^ does not support the notion of a substantial contribution of this intervention to the overall effect.

**Table 2:** Interrupted time series analysis to evaluate changes in the number of physicians, nurses, internists, and surgeons practicing in rural areas, before and after the introduction of the benefits package in 2009

Model component	ARIMA model (p,d,q)	Stationary R2	Estimate (PPC*)	*P* value
Total number of physicians	1.1.0	0.289	620.284	0.295
Total number of nurses	0.2.1	0.269	1197.608	0.585
Total number of internists	0.1.0	0.006	108.722	0.757
Total number of surgeons	0.1.0	0.003	20.444	0.818

*PPC, percentage point change

**Fig. 1:**
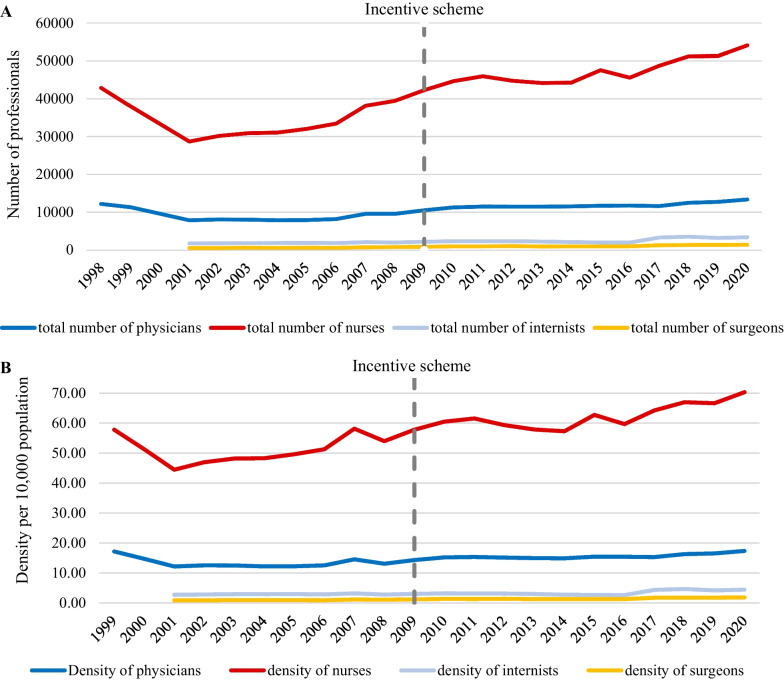
The number (**A**) and density (**B**) of physicians, nurses, internists and surgeons practicing in rural areas, 1998–2020. The gray dashed line shows the intervention (introduction of incentive scheme in 2009)

Figure 1 provides a visual representation of temporal trends in the number and density of healthcare professionals in rural areas from 1998 to 2020. These data complement the findings presented in Table 2, which highlights a significant increase in the number of nurses. Notably, both the number and density of the rural healthcare workforce experienced a decline in the early 2000s, with the lowest levels being recorded in 2001. The exact cause for this effect is not known, but could be attributed to the enduring consequences of the socioeconomic crisis that afflicted the country in the 1990s and persisted until the early 2000s [8].

Table 3 provides insights into the changing trends in the number and per capita density of medical doctors practicing various medical subspecialties following the introduction of an incentive scheme in 2009. All medical subspecialties are listed in descending order based on the percentage point change. The most substantial increase in density was observed in neurosurgeons, followed by cardiologists and trauma-orthopedic specialists. In general, after 2009, the number and density of subspecialists practicing in rural areas increased following the implementation of two national projects ("Construction of 350 Family Health Centers, Feldsher-Obstetric Stations, and Polyclinics," and "Construction of 100 Schools, 100 Hospitals") between 2008 and 2016 to expand the network of healthcare facilities in rural areas [10]. However, during the period under study, the number of pediatricians declined by 13.84%. This trend is alarming, especially considering the high birth rates observed in rural areas of Kazakhstan [21]–[23].

**Table 3:** Variations in the number and density of different types of rural specialists between 2001–2008 and 2009–2020

Type of medical specialty	Mean number/density for the period 2001–2008	Mean number/density for the period 2009–2020	Percentage point change for mean/density
Neurosurgery	1.38/ < 0.01	6.67/0.01	384.85/311.57
Cardiology	38.50/0.06	115.33/0.15	199.57/160.85
Traumatology and Orthopedics	35.25/0.05	96.83/0.13	174.70/139.95
Endocrinology	39.25/0.06	95.33/0.13	142.89/111.54
Radiology	128.50/0.20	308.33/0.41	139.95/107.83
Neurology	143.50/0.22	252.00/0.33	75.61/52.57
Urology	21.25/0.03	37.25/0.05	75.29/52.43
Anesthesiology and Intensive Care	209.50/0.32	328.17/0.43	56.64/36.02
General surgery	380.13/0.58	564.17/0.74	48.42/29.11
Otorhinolaryngology	101.75/0.15	138.50/0.18	36.12/18/27
Ophthalmology	113.13/0.17	153.75/0.20	35.91/18.06
Obstetrics and Gynecology	620.00/0.94	769.67/1.02	24.14/7.68
Dentistry	514.25/0.78	618.83/0.82	20.34/4.30
Internal medicine	1599.25/2.44	1640.33/2.17	2.57/− 10.93
Pediatrics	1454.00/2.22	1252.83/1.66	− 13.84/− 25.15

The comparisons between the total numbers and per capita densities of all internal medicine subspecialties and surgical specialties practicing in rural and urban areas between 2001–2008 and 2009–2020 are presented in Table 4. Both the numbers and per capita densities of physicians were significantly higher for urban areas for all categories of specialists, except for per capita densities of all internal medicine subspecialties. The DID analysis showed that the introduction of the incentive scheme did not have a significant impact on specialists practicing in rural areas.

**Table 4:** Total number and per capita densities of all internal medicine subspecialties and surgical specialties in rural and urban areas between 2001–2008 and 2009–2020

Comparisons between rural and urban physicians		Mean numbers (± SD*)		Mean per capita densities (± SD)	
		2001–2008	2009–2020	2001–2008	2009–2020
All internal medicine subspecialties	Rural	1923.38 (± 110.45)	2623.67 (± 588.22)	2.725 (± 0.27)	3.425 (± 0.72)
	Urban	10692 (± 514.55)	15914.67 (± 4908.63)	12.463 (± 0.54)	15.867 (± 3.73)
Student’s *t* test, *p* value		0.027	< 0.001	0.307	< 0.001
Difference in differences		*β* = 700.292, *p* = 0.579		*β* = 0.700, *p* = 0.473	
All surgical specialties	Rural	658.13 (± 88.84)	1128.17 (± 192.45)	0.988 (± 0.08)	1.483 (± 0.24)
	Urban	5657.38 (± 387.09)	8085.50 (± 2195.90)	6.638 (± 0.43)	8.083 (± 1.59)
Student’s *t* test, *p* value		0.003	< 0.001	< 0.001	< 0.001
Difference in differences		*β* = 470.042, *p* = 0.408		*β* = 0.496, *p* = 0.241	

*SD standard deviation

Table 5 reflects temporal trends in the distribution of the primary healthcare workforce during the period 2009–2020. As the table demonstrates, the number of rural primary practitioners increased over the study period, and correspondingly, so did the proportions of primary practitioners to the total number of physicians and the per capita rates, which by the end of the study period (2020) constituted 26.71% and 4.64 per 10,000 population, respectively.

**Table 5:** Variations in the numbers, proportions, and density of rural primary practitioners, 2009–2020

Year	Total number of rural physicians	Number (%) of primary practitioners	Number of rural population	Density of primary practitioners
2009	10,505	454 (4.32)	7,319,451	0.62
2010	11,254	1712 (15.21)	7,383,654	2.32
2011	11,473	1960 (17.08)	7,466,548	2.63
2012	11,448	2004 (17.51)	7,546,390	2.66
2013	11,451	2049 (17.89)	7,632,375	2.68
2014	11,538	2237 (19.39)	7,727,280	2.89
2015	11,713	2448 (20.90)	7,578,690	3.23
2016	11,767	2579 (21.92)	7,634,319	3.38
2017	11,619	3393 (29.20)	7,586,722	4.47
2018	12,481	3533 (28.31)	7,647,541	4.62
2019	12,736	3567 (28.01)	7,697,359	4.63
2020	13,365	3570 (26.71)	7,693,127	4.64

**Fig. 2:**
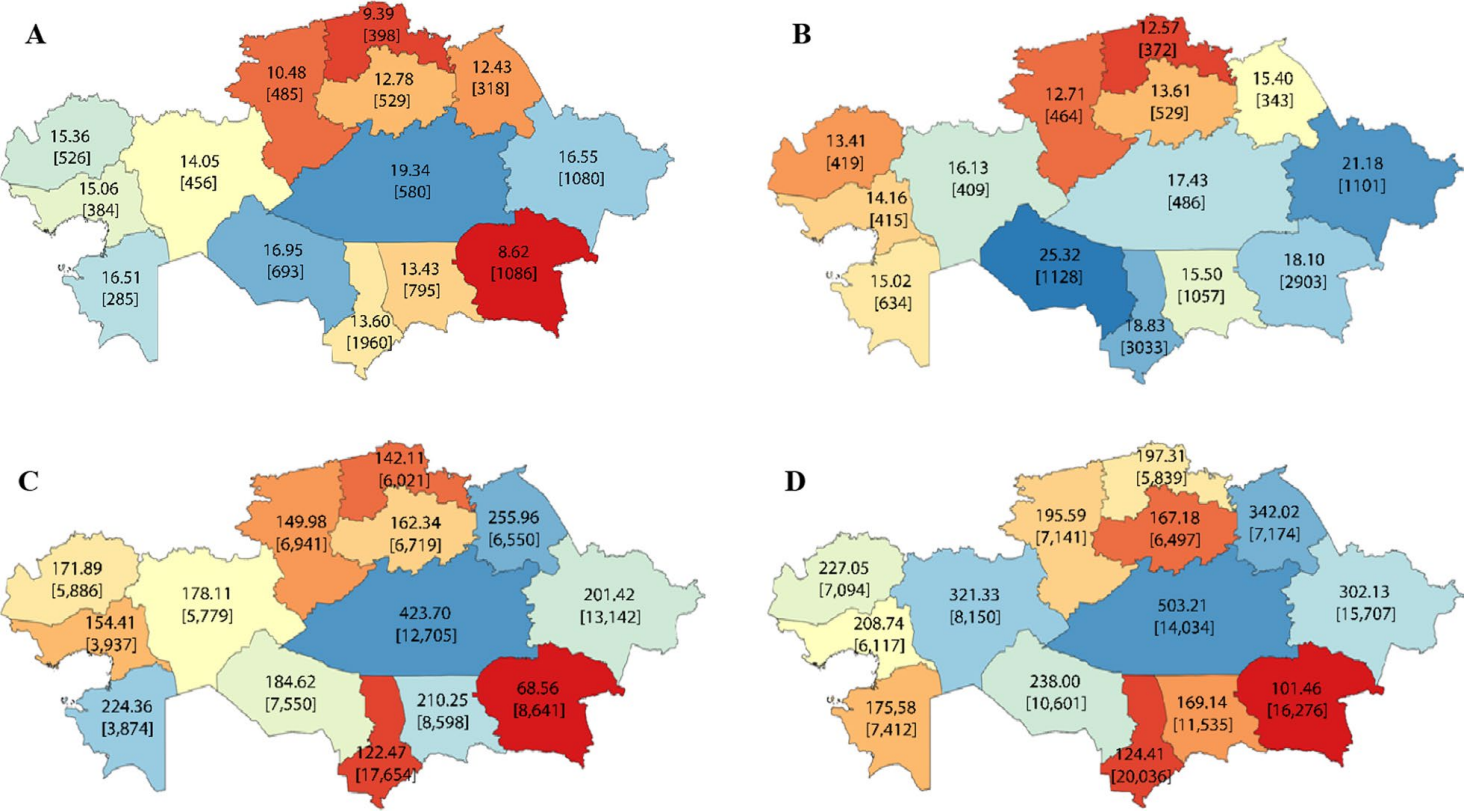
The density [numbers] of rural physicians in 2008 (**A**) and 2020 (**B**), and rural nurses in 2008 (**C**) and 2020 (**D**) by region of Kazakhstan

Figure 2 illustrates the density of rural physicians and nurses in 2008 and 2020. There was considerable variation in the density of both physicians and nurses across regions of Kazakhstan. The Karaganda, East Kazakhstan, and Aktobe regions exhibited higher densities than the national average in both 2008 and 2020. This finding is not surprising, as these regions have their own medical schools.

## Discussion

The study aimed to assess the impact of the national incentive scheme introduced in 2009 on the numbers and density of healthcare personnel in rural areas in Kazakhstan. Additionally, it reports on the temporal and spatial trends in the number and density of the rural healthcare workforce in Kazakhstan from 1998 to 2020. Although there was a significant increase in the number of rural healthcare workers from 2009 to 2020 compared to the period of 1998 to 2008, this increase was less pronounced in per capita terms, and a decline in the density of internists and pediatricians was observed. If this decline continues, it could have an adverse impact on the well-being of the rural population, particularly given the increasing longevity and high birth rates in rural Kazakhstan [9]. An interrupted time series analysis conducted via ARIMA modeling indicated that the initiation of the incentive scheme in 2009 failed to influence the growth trajectory of both the quantity and density of the rural healthcare workforce. Furthermore, a DID analysis utilizing urban physicians as a control group corroborated that the observed increments in the count and density of rural internists and surgeons cannot be attributed to the incentive scheme. These findings require detailed discussion in relation to other studies on the topic.

The density of healthcare professionals in rural areas of Kazakhstan is comparable to that of high-income countries. According to data from the Global Health Observatory, established by the WHO, the density of nursing and midwifery personnel in Australia was 164 per 10,000 population, in Austria it was 107.7, in Finland it was 223.2, and in Japan it was 124.5 in 2020. In contrast, some of the world's poorest nations, such as Afghanistan, Malawi, and Chad, had only 4.52, 7, and 2 nurses per 10,000 population, respectively [13]. It should be noted that the density rates observed for Kazakhstan may be inflated, as they include all types of medical professionals with secondary education. Similarly, the per capita density of rural physicians in Kazakhstan is comparable to that of many middle-income countries. For example, in 2019, Brazil had 23.03 physicians per 10,000 population, Chile had 26.49, and China had 22.58 [13]. However, these data should be considered illustrative, as they represent the general population. Nevertheless, it can be concluded that the per capita density of the rural healthcare workforce in Kazakhstan is adequate globally.

However, in regard to the rural healthcare workforce in Kazakhstan, other factors must be taken into account to determine its adequacy. The primary factor is the significantly low population density in rural areas [4], coupled with the need to cover long distances while traveling from one settlement to another. Moreover, the southern and eastern regions of Kazakhstan comprise mountainous areas that pose additional challenges in terms of geographical access. Given the physical barriers, the number of rural health professionals should not be considered excessive. Instead, it is a common practice in Kazakhstan to arrange a network of healthcare facilities with a professional workforce. The practice of having rural healthcare workers with no facility is becoming obsolete, as evidenced by only eight workers in this category being employed in the Zhambyl region as of 2020 [14]. There are specific regulations in Kazakhstan that determine the density of healthcare facilities, with the latest order from 2020 being very flexible in terms of determining the population size required to open a healthcare facility in rural areas. In fact, medical stations (the smallest healthcare facility in Kazakhstan) can be established in rural settlements with fewer than 50 inhabitants if there is no other healthcare facility within a 5-km radius [24].

There is a dearth of studies addressing the problem of insufficiency of healthcare workforce in rural areas of Kazakhstan. The study by Turgambayeva et al. reported that 83% of all physicians in Kazakhstan practice in urban areas, with only 17% practicing in rural areas, despite the fact that the share of the rural population comprises 41%. Additionally, the authors emphasize that despite many efforts made by the Kazakhstani government to increase the number of physicians working in rural areas, the shortage of medical specialists still exists [25]. Koichubekov et al. agree with this statement [26]. The description of the incentive scheme implemented in Kazakhstan between 2019 and 2022 is presented in the study by Malbekova et al. However, the study lacks analysis of the impact made by this scheme [27].

Globally, recruiting and retaining healthcare personnel in rural areas is a major problem, and this can be explained in various ways. The lack of medical facilities and resources in rural areas makes it difficult for healthcare providers to gain the experience they need to become proficient in their field. Additionally, rural areas often offer lower pay rates than urban areas, making it challenging for healthcare providers to justify living and working in such regions [28]. Another significant issue is the lack of professional development opportunities. In rural areas, healthcare workers may have limited access to continuing education and professional development programs. This can result in a lack of career progression and decreased job satisfaction, ultimately leading to high turnover rates [29]. Furthermore, the isolation and limited social opportunities that come with living and working in rural areas can also hinder the attraction and retention of healthcare workers. Many healthcare professionals, especially those from urban areas, may find it challenging to adjust to the slower pace of life in rural areas [30]. The lack of access to cultural events, restaurants, and other amenities can make it difficult to recruit and retain healthcare professionals. Additionally, the lack of access for the children of healthcare professionals to better schools, cultural, and recreational activities often influences professionals to favor urban areas instead of rural ones [31].

Addressing the scarcity of healthcare workers in rural areas necessitates a multifaceted approach, given its intricate nature. Apart from financial incentives, alternative improvements could be considered. These may include fostering opportunities for professional advancement through the provision of training courses for skill enhancement, forging partnerships with centers of excellence offering attachment opportunities for rural professionals [2]. Moreover, investments in infrastructure and technology are imperative. Equipping rural healthcare facilities with modern equipment serves to attract young professionals [4]. Additionally, it is essential to implement measures that mitigate the challenges encountered by healthcare workers in these regions, such as inadequate social infrastructure and facilities, which adversely impact the well-being of healthcare workers and diminish their inclination to continue practicing in rural areas [6].

The study has both strengths and limitations. It represents the first investigation into the effects of the incentive scheme introduced in 2009 on the number of rural healthcare workers in Kazakhstan, and it is also the first to examine the spatial and temporal trends in the distribution of various types of medical professionals.

The primary strength of the study lies in the availability of a large set of administrative data that covers a period of 23 years (1998–2020). However, the study also has limitations due to the aggregated nature of the data, which were obtained from official statistical compilations issued by the MoH Kazakhstan. For example, the analysis was unable to assess the numbers and density of different nursing personnel since these data were presented in an aggregated manner. Additionally, the data on healthcare workforce and healthcare institutions are contained in different subsections of the official reports, and there is no information available on the density of healthcare workforce per institution. The absence of information on healthcare workforce density per institution hinders the evaluation of workforce shortages per facility type and its impact on rural healthcare access. Furthermore, the study could not examine the incentive scheme's impact on other medical specialties, particularly primary care practitioners, as relevant data were only available from 2009 onward. Besides, they lack disaggregation by the regions of Kazakhstan. Future research should be undertaken to examine the impact made by the incentive scheme on other medical subspecialties, particularly primary practitioners. Given that the interrupted time-series analysis and DID analysis failed to establish a significant association between the introduced incentives and the upsurge in the number of healthcare workers, further investigations with preferably prospective designs are imperative to identify additional contributing factors.

The findings of this study offer valuable insights for policymakers regarding the effectiveness of measures aimed at improving the availability of healthcare workers in rural regions of Kazakhstan. Moreover, they provide valuable considerations for an international audience evaluating the interventions implemented in Kazakhstan and their subsequent effects. However, the findings do not support the continuation of the governmental incentive scheme. To ensure that the governmental incentive scheme has an impact on the rural healthcare workforce, initiating a public discussion to evaluate its composition, duration, and conditions for provision could be beneficial. Overall, while various incentive schemes have been globally implemented to address healthcare workforce shortages and enhance the attractiveness of rural practice, evidence of their effectiveness remains limited.

## Conclusions

The government of Kazakhstan introduced an incentive scheme in 2009, which included a one-time allowance and housing incentive expressed in MCI, to encourage professionals to relocate to rural areas. However, due to the annual revision of the MCI and devaluations of the Kazakhstani Tenge, the dollar equivalent value of the incentive scheme has decreased. It is imperative to instigate public discussion concerning the value and composition of the incentive scheme, as well as the terms of its provision. This is underscored by one of the principal findings of the present study, which indicates that, currently, it appears to exert no discernible impact on the expansion of rural healthcare workforce numbers and densities.

## Data Availability

The datasets used and/or analyzed during the current study are available from the corresponding author on reasonable request.

## Abbreviations

ACF: Autocorrelation
ARIMA: Autoregressive integrated moving average
BNS: Bureau of National Statistics
DID: Difference in Differences
MCI: Monthly Calculation Index
MoH: Ministry of Health
PACF: Partial autocorrelation
PPC: Percentage point change
SPSS: Statistical Package for Social Sciences
USD: United States Dollar
USSR: Union Soviet Socialist Republics
WHO: World Health Organization
